# The Prognosis Predictive Score around Neo Adjuvant Chemotherapy (PPSN) Improves Diagnostic Efficacy in Predicting the Prognosis of Epithelial Ovarian Cancer Patients

**DOI:** 10.3390/cancers15205062

**Published:** 2023-10-19

**Authors:** Naoki Kawahara, Shoichiro Yamanaka, Sumire Sugimoto, Junya Kamibayashi, Kyohei Nishikawa, Ryuji Kawaguchi, Fuminori Kimura

**Affiliations:** Department of Obstetrics and Gynecology, Nara Medical University, 840 Shijo-cho, Kashihara 634-8521, Japan; shoichiroyamanaka@naramed-u.ac.jp (S.Y.); sumire6003@naramed-u.ac.jp (S.S.); junya.0714@naramed-u.ac.jp (J.K.); k196560@naramed-u.ac.jp (K.N.); kawaryu@naramed-u.ac.jp (R.K.); kimurafu@naramed-u.ac.jp (F.K.)

**Keywords:** epithelial ovarian cancer (EOC), neo adjuvant chemotherapy (NACT), interval debulking surgery (IDS), prognosis predictive score around neo adjuvant chemotherapy (PPSN), inflammatory response, progression-free survival (PFS), overall survival (OS), tumor infiltrating lymphocytes (TILs)

## Abstract

**Simple Summary:**

A recent study conducted at our institution aimed to identify factors predicting prognosis for advanced epithelial ovarian cancer (EOC) patients who underwent pre-treatment inflammatory response analysis with pre-treatment and post-neo adjuvant chemotherapy (NACT). The study was conducted between June 2006 and March 2020, with demographic and clinicopathological data collected from 72 patients who underwent NACT followed by interval debulking surgery (IDS). The study created a novel predictive scoring system called the Predictive Prognosis Score around NACT (PPSN) using factors extracted from a receiver operating characteristic curve analysis. The study found that a high PPSN (≥4) significantly predicts poor prognosis, and CD3^+^ and CD8^+^ tumor-infiltrating lymphocytes in those with a low PPSN (<4) showed higher aggregation than those in high PPSN (≥4) cases. The study concluded that PPSN could be a useful prognostic tool for advanced EOC patients who undergo NACT followed by IDS.

**Abstract:**

Background: Recent studies have shown that pretreatment inflammatory responses can predict prognosis. However, no reports have analyzed the combined effect of the inflammatory response with pre-treatment and post-neo adjuvant chemotherapy (NACT). This retrospective study aims to identify factors predicting prognosis and create a novel predictive scoring system. Methods: The study was conducted at our institution between June 2006 and March 2020. Demographic and clinicopathological data were collected from patients with advanced epithelial ovarian cancer who underwent neoadjuvant chemotherapy after sample collection by laparoscopic or laparotomy surgery, followed by interval debulking surgery. We created a scoring system, called the Predictive Prognosis Score around NACT (PPSN), using factors extracted from a receiver operating characteristic curve analysis. Univariate and multivariate analyses were conducted to assess the efficacy of PPSN in predicting progression-free survival and overall survival. Kaplan-Meier and log-rank tests were used to compare the PFS or OS rate. Results: Our study included 72 patients, with a cut-off value of four for the scoring system. Our analysis showed that high PPSN (≥4) significantly predicts poor prognosis. Moreover, CD3^+^ and CD8^+^ tumor-infiltrating lymphocytes with low PPSN (<4) showed higher aggregation than those with high PPSN (≥4) cases. Conclusion: Our study shows that PPSN could be a useful prognostic tool for advanced EOC patients who undergo NACT followed by IDS.

## 1. Introduction

Epithelial ovarian cancer (EOC) is women’s fifth leading cause of cancer-related death in the US [1], and about 200,000 new cases are reported annually worldwide [2,3]. Due to the nature of fewer symptoms of invasive malignant tumors, a large proportion of EOC cases are diagnosed at advanced stages based on the International Federation of Gynecology and Obstetrics (FIGO) (e.g., about 63% at stage IV). The overall survival rate according to the FIGO Stages I, II, and III/IV was reported as 74.5%, 54.5%, and 24.7%, respectively [4]; thus, this disease is called the silent killer [5,6]. The recurrence rate rises according to the FIGO stage and advanced stages as III and IV show a high recurrence rate of approximately 80% [7]. Complete or optimal cytoreductive surgery, defined as grossly no residual cancer or less than 10 mm of residual disease, is known as the most important prognostic factor because the residual tumor is related to a lower progression-free survival (PFS) and overall survival (OS) [8,9]. Thus, the gold standard of EOC treatment is primary debulking surgery (PDS) followed by such maintenance treatment as chemotherapy [10,11]. When the physician encounters a problem in conducting the complete surgery due to a strong invasion of the sigmoid colon and other pelvic organs, neoadjuvant chemotherapy (NACT) and delayed interval debulking surgery (IDS) are alternative options based on perspective and retrospective studies, which showed a similar prognosis compared to PDS, as well as an increased optimal debulking rate, improved quality of life, and decreased surgery-related complications [12,13].

In recent years, inflammatory reactions in the tumor microenvironment have been shown to play an important role in tumor development and progression [14,15]. Peripheral leukocytes, neutrophils, lymphocytes, platelets, and acute-phase proteins contribute to the inflammatory response and can be used as prognosis-predictive factors. These consist of tumor-related leukocytosis (TRL) [16,17], the neutrophil/lymphocyte ratio (NLR) [18,19,20], platelet/lymphocyte ratio (PLR) [21,22], and systemic immune-inflammation index (SII) [23,24,25]. High levels of leukocytes, neutrophils, and platelets are adverse prognostic factors caused by an increase in hematological growth factors such as granulocyte colony-stimulating factor, granulocyte-macrophage colony-stimulating factor, interleukin 1 (IL-1), IL-6, and tumor necrosis factor-alpha (TNF-α). These factors lead to a poor prognosis. On the other hand, lymphocytes play a crucial role in controlling tumor growth by secreting cytokines such as interferon-gamma and TNF-α, which results in a good prognosis. Furthermore, the Glasgow Prognostic Score (GPS) and modified GPS (mGPS) have been found to be reliable prognostic indicators in cancer patients. The scores are based on a combination of serum CRP elevation and decreased albumin concentration, which is indicative of systemic inflammation and can reflect the overall health of the patient. These markers have been shown to be significant in predicting outcomes in patients with cancer [26,27,28].

Currently, there are several indices used to evaluate a patient’s prognosis at a specific point in time. However, there is no predictive scoring system that combines the peripheral blood biomarkers before and after NACT for the PFS or OS. Then we hypothesized that a combination of such factors at pre- and post-treatment points might be more accurate in predicting prognosis than a single-point assessment. This study aims to seek the prognostic factors around NACT in ovarian cancer and create the prognostic score predicting the prognosis of EOC.

## 2. Materials and Methods

### 2.1. Patients

A list of patients with primary, previously untreated, histologically-confirmed ovarian cancers who were treated at Nara Medical University Hospital between June 2006 and March 2020 was generated from our institutional registry. A total of 142 cases who underwent tumor biopsy were eligible. A total of 73 cases who underwent IDS were extracted. Then, one case was excluded because of the missing data. Thus, a total of 72 patients were included in the current cohort. Paclitaxel and carboplatin chemotherapy were primarily used as NACT in 71 out of 72 cases. Other regimens were started or substituted in the event of allergy or other issues. All cases were histologically confirmed. Written consent for the use of the patient’s clinical data for research was obtained at the first hospitalization, and after approval by the Ethics Review Committee of the Nara Medical Hospital, the opt-out form was provided through our institutional homepage. No patients had undergone chemotherapy or radiotherapy for ovarian tumors before treatment.

The inclusion criteria were as follows: (I) epithelial ovarian cancer which could not be removed by primary surgery due to the invasions or metastasis to other organs; (II) received neoadjuvant chemotherapy with interval debulking surgery. The exclusion criteria were as follows: (I) combined with other malignant tumors; (II) metastatic ovarian cancer or postoperative recurrence of ovarian cancer; (III) patients who received other treatment, such as immunotherapy.

### 2.2. Collection of Candidates Predicting Mortality

The following factors were collected through a chart review of the patient’s medical records: age, body mass index (BMI), parity, postoperative diagnosis including FIGO stage, TNM classifications, tumor subtypes, surgical outcomes, and pre-treatment and post-NACT blood test results. Pre-treatment blood samples were obtained at the first visit to our hospital and a post-NACT blood test was conducted on the outpatient visit for IDS. The difference between pre-treatment and post-NACT was also analyzed, which is calculated by subtraction of the pretreatment value from the post-NACT.

### 2.3. Statistical Analysis

Analyses were performed using SPSS version 25.0 (IBM SPSS, Armonk, NY, USA). The differences in each factor were compared using a Student *t*-test or a Mann–Whitney U test after assessing whether normal distribution or not. The receiver operating characteristic (ROC) curve analysis was performed to determine the cut-off value for predicting poor prognosis. The cut-off value was based on the highest Youden index (i.e., sensitivity + specificity − 1). We next used a logistic regression analysis to assess the risk factors for mortality. A two-sided *p* < 0.05 was considered as indicating a statistically significant difference.

### 2.4. Tumor Infiltrating Lymphocytes (TILs) Assessment

Four paraffin sections of serial 3 μm thickness were taken from each original block which was obtained by the first-looking surgery, one section was stained with hematoxylin and eosin for diagnostic confirmation and the other three sections were immunostained for CD3, CD8, and CD56 by immuno-enzyme polymer methods and conventional methods, respectively, using an avidin–biotin complex immunoperoxidase technique. These tissue specimens were immunostained using the Leica BONDMAX systems (Mitsubishi Chemical Medicine Co., Tokyo, Japan). The primary antibodies used in this study were: CD3 mouse monoclonal antibody (Clone F7.2.38, Dako, CA, USA; 1:100 dilution), CD8 mouse monoclonal antibody (Clone 4B11, Leica Biosystems, Newcastle, UK; 1:1 dilution), and CD56 mouse monoclonal antibody (Clone 1B6, Leica Biosystems, Newcastle, UK; 1:100 dilution). TILs and sTILs evaluations were performed on hematoxylin and eosin (HES)-stained whole sections of either formalin- or acetic acid formalin alcohol (AFA)-fixed tissue. TILs and sTILs were defined as the summary of lymphocyte numbers on 400 times fields for 20 fields which were randomly selected by S.S. as a pathologist. TILs and sTILs were assessed by the two researchers including one pathologist (S.S.) completely blinded to the outcome data and the results were averaged.

### 2.5. Checklist

The current study was described according to STROBE (strobe-statement.org) checklist in reports of cohort studies.

## 3. Results

### 3.1. Patients

From June 2006 and March 2020, a total of 72 patients were included in this study. Patients’ peripheral blood data were collected before biopsy (or NACT) and IDS, and the median number of days between the start of NACT and IDS was 144 days, and between blood test visits after NACT and IDS it was 15 days. The mortality rate was 61.1% (44 cases). The demographic and clinical characteristics of the current cohort are outlined in Table 1.

The serous cases were high-grade serous carcinoma. There was no significant differentiation between dead and live cases in the background. The median days for the first treatment and IDS showed significant differentiation between the live and dead cases (111.0 (83–234) vs., 147.0 (85–284), *p* = 0.002, respectively).

Pre-treatment analysis of peripheral blood cells and serum markers is demonstrated in Supplementary Table S1. In the current cohort, neutrophil (%), lymphocyte (%), and lymphocyte counts showed significant differentiation in the pretreatment distribution of peripheral blood cells. An analysis of peripheral blood cells and serum markers after NACT (before the IDS) is shown in Supplementary Table S2. The post-NACT platelet counts with the factors extracted in the pre-treatment analysis showed significant differentiation.

### 3.2. The Candidates Predicting the Mortality of Ovarian Cancer

**Figure 1:**
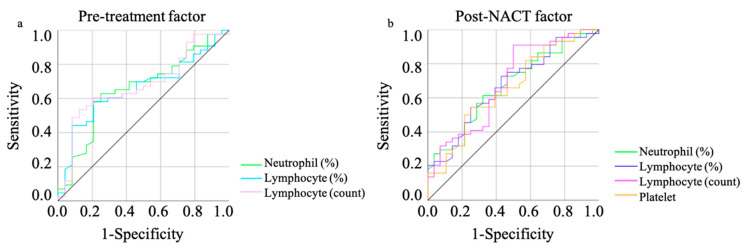
The result of the ROC curve analysis based on predicting mortality including pre-treatment (**a**) and post-NACT (**b**) factors. ROC: receiver operating characteristic, NACT: neo adjuvant chemotherapy.

The results of the receiver operating characteristic (ROC) curve analysis based on the mortality are shown in Table 2. The optimal cutoff values were determined by analyzing the ROC curve predicting the mortality (Figure 1).

The ROC analysis significantly showed that neutrophil (%), lymphocyte (%), and lymphocyte counts in both pre-treatment and post-NACT, and platelet counts after NACT, showed efficacy (Table 2). Multivariate analysis showed that lymphocyte counts in the pre-treatment and after NACT treatment were the independent prognostic factor (hazard ratio [HR]: 5.71, 95% confidence interval (CI): 1.38–23.67, *p* = 0.016; HR: 6.94, 95% CI: 1.76–27.33, *p* = 0.006, respectively). The FIGO stage did not show efficacy in predicting mortality (Table 3).

### 3.3. The Efficacy of Prognosis Predictive Score around NAC (PPSN)

**Figure 2:**
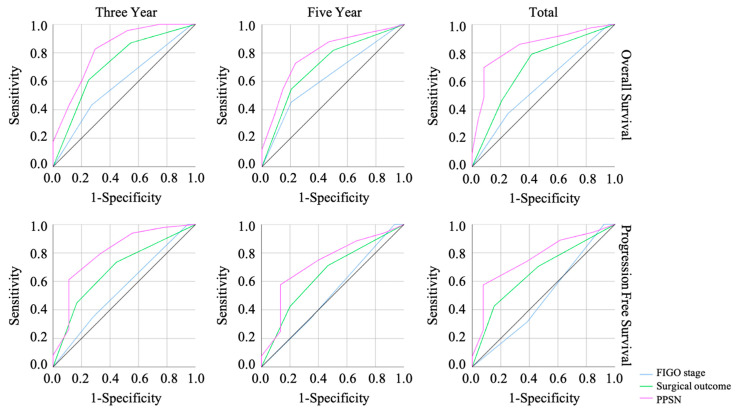
The result of the ROC curve analysis based on the FIGO stage, surgical outcomes, and PPSN in three-year, five-year, and total OS and PFS. PPSN showed the outstanding effect in predicting prognosis. ROC: receiver operating characteristic, OS: overall survival, PFS: progression-free survival, PPSN: prognosis predictive score around neo adjuvant chemotherapy.

PPSN is defined by neutrophil (%), lymphocyte (%), and lymphocyte counts in both pre-treatment and post-NACT, and platelet counts after NACT; if all parameters are abnormal, the assigned value is seven; and if all parameters are normal, the assigned value is zero. Elevated proportion and counts of neutrophils, decreased proportion and counts of lymphocytes, and decreased platelet counts are considered abnormal parameters. The ROC analysis showed that the PPSN was the outstanding method to predict not only the overall survival (OS) but also the progression-free survival (Figure 2).

It is a well-known fact that the complete resection of residual tumors in advanced ovarian cancer improves prognosis [8,9]. We analyzed the prognosis efficacy of PPSN including the FIGO stage and the residual tumor size. Multivariate analysis also showed that the PPSN was the independent prognostic factor in predicting the mortality in three-year, five-year, and total OS (hazard ratio [HR]: 11.32, 95% confidence interval (CI): 3.22–39.84, *p* < 0.001; HR: 8.66, 95% CI: 2.87–26.09, *p* < 0.001; HR: 25.38, 95% CI: 5.19–124.11, *p* < 0.001, respectively) (Table 4).

**Figure 3:**
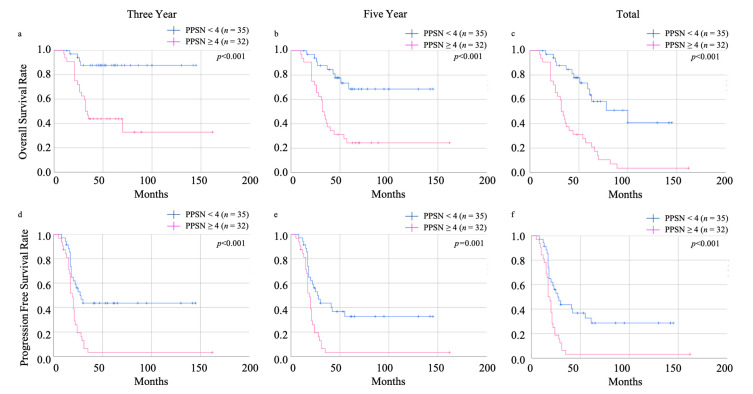
Log lank analysis showed that low PPSN scores (<4) indicated good prognosis in three-year (**a**), five-year (**b**), and total (**c**) OS (all were *p* < 0.001), and three-year (**d**), five-year (**e**), and total (**f**) PFS (*p* < 0.001; *p* = 0.001; *p* < 0.001, respectively). PPSN: prognosis predictive score around neo adjuvant chemotherapy, PFS: progression-free survival, OS: overall survival.

The scoring system also revealed effectiveness in discriminating between non-recurrent and recurrent cases in three-year, five-year, and total PFS (HR: 12.63, 95% CI: 2.60–61.22, *p* = 0.002; HR: 8.86, 95% CI: 1.81–43.33, *p* = 0.007; HR: 16.17, 95% CI: 1.96–133.42, *p* = 0.010, respectively) (Supplementary Table S3). Log lank analysis revealed that low PPSN scores (<4) showed a good prognostic efficacy in three-year, five-year, and total OS (all were *p* < 0.001) (Figure 3). Three-year, five-year, and total PFS also showed a good prognosis efficacy (*p* < 0.001; *p* = 0.001; *p* < 0.001, respectively) (Figure 3).

### 3.4. Peripheral Blood Lymphocyte Counts in the Pre-Treatment and Post-NACT Points Correlate with the TILs Distribution

Among these factors consisting of PPSN, the lymphocyte counts both of pre- and post-NACT show a dominant role in predicting the prognosis; thus, we hypothesized that the lower PPSN case has a larger lymphocyte load into the tumor or stromal area. Table 5 showed the distribution of tumor-infiltrating lymphocytes (TILs) or stromal tumor-infiltrating lymphocytes (sTILs) according to PPSN scoring.

**Figure 4:**
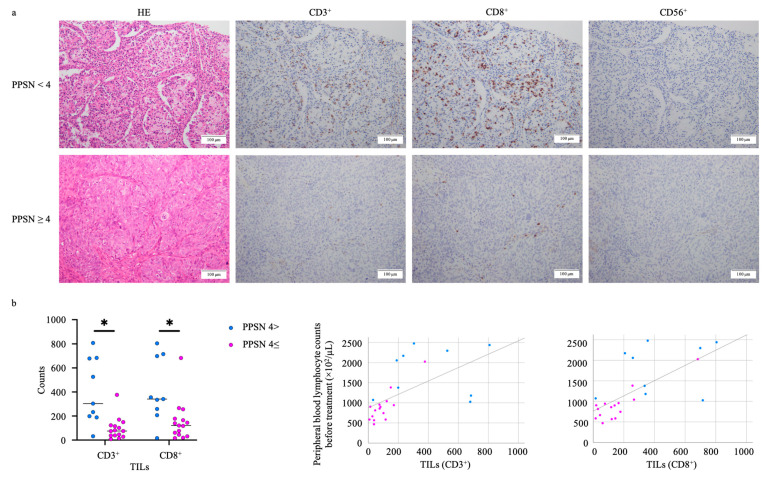
(**a**) Immunohistochemical expression status of CD3, CD8, and CD56 in samples of low PPSN (<4) and high PPSN (≥4). TILs and sTILs evaluations were performed on hemoxylin and eosin (HES)-stained whole sections. (**b**) The CD3^+^ and CD8^+^ TILs with the high PPSN cases showed a significantly higher distribution than with the low (*p* = 0.001 and 0.006, respectively). The CD3^+^ and CD8^+^ TILs showed a significant correlation with peripheral blood lymphocyte counts (*p* = 0.002 and *p* = 0.006, respectively). TILs: tumor-infiltrating lymphocytes. PPSN: prognosis predictive score around neo adjuvant chemotherapy. * *p* < 0.05.

The CD3^+^ and CD8^+^ TILs also showed significant differences between high and low PPSN scores (*p* = 0.001 and 0.006, respectively). Moreover, CD3^+^ and CD8^+^ TILs correlated with peripheral blood lymphocyte counts (*p* = 0.002 and *p* = 0.006, respectively) (Figure 4).

## 4. Discussion

Among several studies reported as predictive tools for PFS and OS in advanced EOC, at least to our knowledge, there is no prognostic scoring system taking into account pre- and post-NACT patients’ data. We reported the scoring system named the prognosis predictive score around primary debulking surgery (PPSP) for progression-free survival (PFS), in which there were no patients shared in the current cohort [29]. This scoring system is effective to predict the patient’s prognosis for those who underwent primary debulking surgery. But there are a large number of patients who cannot undergo the primary debulking surgery when the tumor invades other pelvic organs such as the small intestine, colon, or bladder. In this case, the physician conducts only sampling followed by NACT and this leads to the IDS. The current study revealed that the PPSN showed great efficacy in predicting not only the mortality but also the progression of the EOC cases who underwent the IDS.

There was a confounding bias in the differentiation of the NACT cycle between live and dead cases, which should lead to the residual tumor size. In clinical settings, the physician decides the cycle of chemotherapy according to the tumor size reduction or peripheral blood tumor markers, and it is assumed that the dead cases should show a relatively poor response to chemotherapy. By the multivariate analysis, the PPSN was elucidated as a better prognostic factor than the residual tumor status after IDS. This might be a crucial tool to predict the prognosis of advanced EOC cases.

This current study showed that the elevated pre-treatment lymphocyte counts in the peripheral blood could contribute to good OS and PFS, comparable to previously reported evidence (e.g., NLR and MLR) [18,19,20,30]. Moreover, we demonstrated that elevated post-NACT lymphocyte counts could also predict the OS and PFS, by which lymphocytes could have an important role in the tumor microenvironment through the treatment. However, contrary to the previous reports [18,19,20,21,22,23,24,25], the reduced platelet count of post-NACT proved to contribute to poor prognosis. This result should suggest that too-low platelet levels are, conversely, a poor prognostic factor.

We present the impact of the elevated lymphocyte count in the peripheral blood on the lymphocytic infiltrate into the tumor in patients with EOC. The human immune system naturally generates adaptive immune responses against EOC, which has a moderately high mutational load on average, and immune recognition of cancer mutations has previously been shown [31,32]. There has been increasing evidence that the host immune system has a role in controlling cancer growth, and tumor-infiltrating lymphocytes (TILs) have been repeatedly associated with improved survival in EOC [33,34,35,36,37,38,39,40]. We confirmed that increased sTILs supplied by the peripheral bloodstream before the IDS increased the sensitivity of malignant cells and showed a tumor-suppressive effect. Our result could be an important suggestion to select the more aggressive strategy like the longer adjuvant chemotherapy or close follow-up after the chemotherapy.

This study has some limitations. The first limitation is that the current study had a relatively small sample size as a nature of secondary surgery. Second, there would be a potential bias according to the TILs counting by a single pathologist. Efforts were made to avoid bias by performing measurements under completely blinded conditions. Third, we could not elucidate the mechanism of how too low a platelet count could have a negative influence on prognosis in EOC patients. Finally, we did not analyze such serum cytokines as IFN-gamma, IL-2, IL-15, and IL-12, which can increase tumor cell class I MHC expression and sensitivity to lysis by CTLs or tumoricidal capacity of NK cells.

## 5. Conclusions

In conclusion, the PPSN can predict both the PFS and OS for ovarian cancer patients who underwent IDS following NACT.

## Figures and Tables

**Table 1:** Demographic and clinical characteristics of the current cohort.

Demographic or Characteristics	Live	Dead	*p*-Value
Number	*n* = 28	*n* = 44	
Age (years)			
Median (range)	61.00 (38–76)	62.00 (31–77)	
Mean ± SD	61.96 ± 8.49	60.27 ± 11.09	0.707
BMI			
Median (range)	21.70 (15.54–30.70)	21.44 (15.40–31.60)	
Mean ± SD	21.92 ± 3.71	21.93 ± 3.28	0.934
Parity	*^1^		
0	2	8	
≥1	25	36	0.182
FIGO Stage			
II	1	0	
III	21	28	
IV	6	16	0.184
Tumor Subtype			
Serous	23	32	
Endometrioid	0	4 *^2^	
Clear Cell Carcinoma	0	2	
Mucinous	1	1	
Mixed Type	0	1	
Others	4	4	0.443

BMI: body mass index, FIGO: The International Federation of Gynecology and Obstetrics, *^1^ one case missing. *^2^ three cases were Grade 3 and one was Grade 2.

**Table 2:** The cut-off values predicting overall survival.

Index	AUC	*p*-Value	Cut-Off Value	Sensitivity	Specificity	PPV	NPV
Pre-treatment							
Neutrophil (%)	0.655	0.036	73.40	0.628	0.750	81.81	52.94
Lymphocyte (%)	0.657	0.034	14.15	0.442	0.917	90.47	47.82
Lymphocyte (×10^2^/µL)	0.677	0.017	10.67	0.535	0.875	88.46	51.21
Post-NACT							
Neutrophil (%)	0.669	0.016	47.65	0.614	0.679	75.00	52.77
Lymphocyte (%)	0.672	0.015	41.35	0.727	0.571	72.72	57.14
Lymphocyte (×10^2^/µL)	0.688	0.007	16.84	0.909	0.500	74.07	77.77
Platelet (×10^4^/µL)	0.657	0.026	17.35	0.545	0.750	77.41	51.21

NACT: neo adjuvant chemotherapy, PPV: positive predictive value, NPV: negative predictive value, AUC: area under curve.

**Table 3:** Univariate and multivariable analysis predicting the mortality.

Variables	Cut-Offs	Univariate Analysis		Multivariate Analysis	
		Risk Ratio (95% CI)	*p*-Value	Risk Ratio (95% CI)	*p*-Value
FIGO stage	≤3	1.00 (referent)			
	4	2.09 (0.70–6.24)	0.184		
Neutrophil (%) (Pre-treatment)	<73.40	1.00 (referent)			
	≥73.40	5.06 (1.66–15.38)	0.004		
Lymphocyte (%) (Pre-treatment)	≥14.20	1.00 (referent)			
	<14.20	8.70 (1.81–41.76)	0.007		
Lymphocyte (×10^2^/µL) (Pre-treatment)	>10.67	1.00 (referent)		1.00 (referent)	
	≤10.67	8.05 (2.08–31.05)	0.002	5.71 (1.38–23.67)	0.016
Neutrophil (%) (Post-NACT)	<47.70	1.00 (referent)			
	≥47.70	3.35 (1.23–9.10)	0.018		
Lymphocyte (%) (Post-NACT)	>41.40	1.00 (referent)			
	≤41.40	3.55 (1.30–9.66)	0.013		
Lymphocyte (×10^2^/µL) (Post-NACT)	>16.84	1.00 (referent)		1.00 (referent)	
	≤16.84	10.00 (2.81–35.50)	<0.001	6.94 (1.76–27.33)	0.006
Platelet (×10^4^/µL) (post-NACT)	≥17.40	1.00 (referent)			
	<17.40	3.60 (1.27–10.19)	0.016		

FIGO: The International Federation of Gynecology and Obstetrics, NACT: neo adjuvant chemotherapy.

**Table 4:** Univariate and multivariable analysis for the OS including the post-IDS outcome.

Variables	Cut-Offs	Univariate Analysis		Multivariate Analysis	
		Risk Ratio (95% CI)	*p*-Value	Risk Ratio (95% CI)	*p*-Value
3 year OS					
FIGO stage	≤3	1.00 (referent)			
	4	2.14 (0.75–6.07)	0.152		
Surgical outcome	Complete *^1^	1.00 (referent)			
	Others *^2^	4.23 (1.25–14.25)	0.020		
PPSN	<4	1.00 (referent)		1.00 (referent)	
	≥4	11.32 (3.22–39.84)	<0.001	11.32 (3.22–39.84)	<0.001
5 year OS					
FIGO stage	≤3	1.00 (referent)			
	4	3.49 (1.20–10.12)	0.021		
Surgical outcome	Complete *^1^	1.00 (referent)			
	Others *^2^	3.85 (1.35–10.98)	0.011		
PPSN	<4	1.00 (referent)		1.00 (referent)	
	≥4	8.66 (2.87–26.09)	<0.001	8.66 (2.87–26.09)	<0.001
Total OS					
FIGO stage	≤3	1.00 (referent)			
	4	2.09 (0.70–6.24)	0.184		
Surgical outcome	Complete *^1^	1.00 (referent)			
	Others *^2^	4.53 (1.62–12.67)	0.004		
PPSN	<4	1.00 (referent)		1.00 (referent)	
	≥4	25.38 (5.19–124.11)	<0.001	25.38 (5.19–124.11)	<0.001

IDS: interval debulking surgery, FIGO: The International Federation of Gynecology and Obstetrics, PPSN: predictive prognosis score around neo adjuvant chemotherapy. *^1^ Complete means no residual tumor, *^2^ Others means <1 cm or >1 cm of residual tumor.

**Table 5:** Analysis of lymphocyte phenotype in the tissue before neo adjuvant chemotherapy.

PPSN	<4	≥4	*p*-Value
Number	*n* = 9	*n* = 15	
CD3^+^ TILs			
Median (range)	304.00 (32.00–807.50)	75.50 (8.00–376.50)	
Mean ± SD	405.83 ± 273.17	95.83 ± 91.66	0.001
CD3^+^ sTILs			
Median (range)	409.50 (74.00–740.50)	231.50 (18.50–637.00)	
Mean ± SD	408.94 ± 188.75	275.16 ± 175.73	0.108
CD8^+^ TILs			
Median (range)	341.50 (16.00–804.50)	121.00 (16.00–682.50)	
Mean ± SD	414.66 ± 265.58	154.63 ± 165.74	0.006
CD8^+^ sTILs			
Median (range)	348.50 (174.00–976.50)	300.50 (29.50–649.50)	
Mean ± SD	446.72 ± 244.98	291.90 ± 174.03	0.155
CD56^+^ TILs			
Median (range)	1.00 (0.00–3.00)	1.50 (0.00–12.00)	
Mean ± SD	1.11 ± 1.24	2.50 ± 3.49	0.516
CD56^+^ sTILs			
Median (range)	0.00 (0.00–13.50)	2.50 (0.00–25.00)	
Mean ± SD	3.22 ± 5.35	6.00 ± 6.87	0.151

PPSN: prognosis predictive score around neo adjuvant chemotherapy, CD: cluster of differentiation, TILs: tumor infiltrating lymphocytes, sTILs: stromal tumor infiltrating lymphocytes.

## Data Availability

The data presented in this study are available on request from the corresponding author.

## Supplementary Materials

The following supporting information can be downloaded at: https://www.mdpi.com/article/10.3390/cancers15205062/s1, Table S1: Pre-treatment distributions of peripheral blood analysis.; Table S2: Analysis of peripheral blood distributions after neo adjuvant chemotherapy.; Table S3: Univariate and multivariable analysis for the PFS including the post-IDS outcome.
