# Supplementary material for: The Prognosis Predictive Score around Neo Adjuvant Chemotherapy (PPSN) Improves Diagnostic Efficacy in Predicting the Prognosis of Epithelial Ovarian Cancer Patients
Source: Cancers (Basel). 2023 Oct 19;15(20):5062. doi: 10.3390/cancers15205062 (PMC10605019; doi:10.3390/cancers15205062)
Supplement: Supplementary file 1 [file cancers-15-05062-s001.zip › cancers-2563776-supplementary.docx]

| **Index** | **Live** | **Death** | ***p*-Value** |
| --- | --- | --- | --- |
| Number | *n* = 28 | *n* = 44 |  |
| Hb (g/mL) |  |  |  |
| Median (range) | 12.40 (8.60–14.10) | 12.20 (7.20–14.10) |  |
| Mean ± SD | 11.88 ± 1.41 | 11.78 ± 1.53 | 0.807 |
| Platelet (×10^4^/µL) |  |  |  |
| Median (range) | 39.75 (17.40–65.50) | 36.45 (18.90–67.80) |  |
| Mean ± SD | 40.13 ± 12.55 | 37.32 ± 11.72 | 0.338^†^ |
| WBC (×10^2^/µL) |  |  |  |
| Median (range) | 68.00 (33.00–1.34×10^2^) | 73.50 (45.00–1.42×10^2^) |  |
| Mean ± SD | 73.07 ± 18.76 | 75.31 ± 19.20 | 0.694 |
| Neutrophil (%) | ^*1^ | ^*2^ |  |
| Median (range) | 70.05 (53.40–82.7) | 75.40 (50.90–90.80) |  |
| Mean ± SD | 69.34 ± 7.97 | 73.77 ± 8.19 | 0.036^†^ |
| Neutrophil (×10^2^/µL) | ^*1^ | ^*2^ |  |
| Median (range) | 48.68 (31.00–96.34) | 51.09 (28.12–1.28×10^2^) |  |
| Mean ± SD | 51.97 ± 15.37 | 56.53 ± 19.06 | 0.307 |
| Lymphocyte (%) | ^*1^ | ^*2^ |  |
| Median (range) | 19.65 (7.70–35.40) | 15.70 (3.30–32.70) |  |
| Mean ± SD | 20.66 ± 6.38 | 16.91 ± 7.16 | 0.037^†^ |
| Lymphocyte (×10^2^/µL) | ^*1^ | ^*2^ |  |
| Median (range) | 14.13 (5.00–24.78) | 10.41 (4.68–29.04) |  |
| Mean ± SD | 15.22 ± 5.35 | 12.28 ± 5.30 | 0.016 |
| Monocyte (%) | ^*1^ | ^*2^ |  |
| Median (range) | 6.55 (4.70–10.60) | 6.50 (3.70–11.90) |  |
| Mean ± SD | 6.77 ± 1.63 | 6.69 ± 1.87 | 0.855 |
| Monocyte (×10^2^/µL) | ^*1^ | ^*2^ |  |
| Median (range) | 4.83 (3.08–9.22) | 5.13 (2.76–7.56) |  |
| Mean ± SD | 4.96 ± 1.42 | 4.92 ± 1.35 | 0.738 |
| CA125 (×10^2^U/mL) |  |  |  |
| Median (range) | 15.31 (0.98–8.60×10^2^) | 10.42 (0.28–1.79×10^2^) |  |
| Mean ± SD | 58.95 ± 1.62×10^2^ | 25.82 ± 41.62 | 0.554 |
| CRP (mg/dL) |  | ^*2^ |  |
| Median (range) | 1.22 (0.00–12.01) | 3.10 (0.00–10.50) |  |
| Mean ± SD | 2.50 ± 3.02 | 3.21 ± 2.56 | 0.079 |
| Albumin (g/dL) | ^*2^ | ^*2^ |  |
| Median (range) | 3.80 (2.80–4.70) | 3.70 (1.7–7.0) |  |
| Mean ± SD | 3.80 ± 0.56 | 3.76 ± 0.74 | 0.633 |
| D-dimer (µg/mL) | ^*3^ | ^*4^ |  |
| Median (range) | 6.80 (0.90–23.40) | 6.00 (0.90–35.6) |  |
| Mean ± SD | 7.15 ± 5.62 | 8.40 ± 7.95 | 0.709 |

**Table S1.** Pre-treatment distributions of peripheral blood analysis.

Hb hemoglobin, WBC white blood cells, CA125 carbohydrate antigen125, CRP C-reactive protein, ^*1^four cases missing, ^*2^one case missing, ^*3^seven cases missing, ^*4^three cases missing. ^†^calculated by student t-test.

**Table S2.** Analysis of peripheral blood distributions after neo adjuvant chemotherapy.

Hb hemoglobin, WBC white blood cells, CA125 carbohydrate antigen125, CRP C-reactive protein, ^*1^one case missing, ^*2^three cases missing, ^*3^five cases missing. ^†^calculated by student t-test.

| **Index** | **Live** | **Death** | ***p*-Value** |
| --- | --- | --- | --- |
| Number | *n* = 28 | *n* = 44 |  |
| Hb (g/mL) |  |  |  |
| Median (range) | 10.50 (8.20–13.40) | 10.40 (7.40–13.10) |  |
| Mean ± SD | 10.48 ± 1.22 | 10.54 ± 1.19 | 0.838^†^ |
| Platelet (×10^4^/µL) |  |  |  |
| Median (range) | 21.45 (11.60–38.10) | 16.30 (7.30–36.40) |  |
| Mean ± SD | 23.10 ± 8.36 | 18.63 ± 7.25 | 0.025 |
| WBC (×10^2^/µL) |  |  |  |
| Median (range) | 35.50 (17.00–68.00) | 37.00 (15.00–59.00) |  |
| Mean ± SD | 38.25 ± 13.84 | 37.25 ± 11.32 | 0.739^†^ |
| Neutrophil (%) |  |  |  |
| Median (range) | 43.70 (11.00–64.20) | 52.20 (14.40–73.80) |  |
| Mean ± SD | 42.70 ± 13.34 | 51.10 ± 13.03 | 0.010^†^ |
| Neutrophil (×10^2^/µL) |  |  |  |
| Median (range) | 15.28 (1.87–39.64) | 18.96 (3.69–43.54) |  |
| Mean ± SD | 17.49 ± 10.26 | 19.97 ± 9.57 | 0.229 |
| Lymphocyte (%) |  |  |  |
| Median (range) | 43.00 (28.00–66.60) | 37.35 (15.20–72.10) |  |
| Mean ± SD | 43.90 ± 11.01 | 36.83 ± 11.07 | 0.010^†^ |
| Lymphocyte (×10^2^/µL) |  |  |  |
| Median (range) | 16.35 (8.55–24.05) | 12.94 (5.92–21.60) |  |
| Mean ± SD | 15.82 ± 4.08 | 12.97 ± 3.55 | 0.003^†^ |
| Monocyte (%) |  |  |  |
| Median (range) | 9.70 (2.30–24.50) | 9.45 (1.50–23.80) |  |
| Mean ± SD | 10.82 ± 4.71 | 10.08 ± 4.83 | 0.507 |
| Monocyte (×10^2^/µL) |  |  |  |
| Median (range) | 3.61 (0.43–8.10) | 3.53 (0.27–6.90) |  |
| Mean ± SD | 3.95 ± 1.66 | 3.56 ± 1.41 | 0.283^†^ |
| CA125 (U/mL) | ^*1^ |  |  |
| Median (range) | 13.00 (2.00–9.33×10^2^) | 15.00 (4.00–7.66×10^2^) |  |
| Mean ± SD | 60.00 ± 1.78×10^2^ | 66.80 ± 1.58 | 0.834 |
| CRP (mg/dL) |  |  |  |
| Median (range) | 0.10 (0.00–0.83) | 0.10 (0.00–0.80) |  |
| Mean ± SD | 0.11 ± 0.18 | 0.14 ± 0.15 | 0.067 |
| Albumin (g/dL) | ^*2^ |  |  |
| Median (range) | 4.20 (3.50–4.80) | 4.20 (3.50–4.80) |  |
| Mean ± SD | 4.21 ± 0.26 | 4.16 ± 0.27 | 0.488^†^ |
| D-dimer (µg/mL) | ^*3^ |  |  |
| Median (range) | 0.90 (0.50–11.10) | 0.95 (0.40–5.30) |  |
| Mean ± SD | 1.53 ± 2.29 | 1.30 ± 1.05 | 0.647 |

**Table S3.** Univariate and Multivariable analysis for the PFS including the post-IDS outcome.

| **Variables** | **Cut-offs** | **Univariate Analysis** | | **Multivariate Analysis** | |
| --- | --- | --- | --- | --- | --- |
|  |  | **Risk Ratio (95% CI)** | ***p*-Value** | **Risk Ratio (95% CI)** | ***p*-Value** |
| 3 year PFS | | | | | |
| FIGO stage | ≤3 | 1.00 (referent) |  |  |  |
|  | 4 | 1.32 (0.40–4.27) | 0.641 |  |  |
| Surgical  outcome | Complete^*1^ | 1.00 (referent) |  |  |  |
|  | Others^*2^ | 2.56 (0.87–7.52) | 0.085 |  |  |
| PPSN | <4 | 1.00 (referent) |  | 1.00 (referent) |  |
|  | ≥4 | 12.63 (2.60–61.22) | 0.002 | 12.63 (2.60–61.22) | 0.002 |
| 5 year PFS | | | | | |
| FIGO stage | ≤3 | 1.00 (referent) |  |  |  |
|  | 4 | 0.95 (0.28–3.18) | 0.945 |  |  |
| Surgical  outcome | Complete^*1^ | 1.00 (referent) |  |  |  |
|  | Others^*2^ | 2.11 (0.68–6.52) | 0.195 |  |  |
| PPSN | <4 | 1.00 (referent) |  | 1.00 (referent) |  |
|  | ≥4 | 8.86 (1.81–43.33) | 0.007 | 8.86 (1.81–43.33) | 0.007 |
| Total PFS | | | | | |
| FIGO stage | ≤3 | 1.00 (referent) |  |  |  |
|  | 4 | 0.74 (0.21–2.55) | 0.641 |  |  |
| Surgical  outcome | Complete^*1^ | 1.00 (referent) |  |  |  |
|  | Others^*2^ | 2.05 (0.62–6.69) | 0.233 |  |  |
| PPSN | <4 | 1.00 (referent) |  | 1.00 (referent) |  |
|  | ≥4 | 16.17 (1.96–133.42) | 0.010 | 16.17 (1.96–133.42) | 0.010 |

IDS interval debulking surgery, PFS progression free survival, FIGO The International Federation of Gynecology and Obstetrics, PPSN predictive prognosis score around neo adjuvant chemotherapy. ^*1^Complete means no residual tumor. ^*2^Others mean <1cm or >1cm of residual tumor.
